# Vitamin C Content in Fruits: Biosynthesis and Regulation

**DOI:** 10.3389/fpls.2018.02006

**Published:** 2019-01-24

**Authors:** Mario Fenech, Iraida Amaya, Victoriano Valpuesta, Miguel A. Botella

**Affiliations:** ^1^Departamento de Biología Molecular y Bioquímica, Instituto de Hortofruticultura Subtropical y Mediterránea (IHSM), Consejo Superior de Investigaciones Científicas, Universidad de Málaga, Málaga, Spain; ^2^Instituto Andaluz de Investigación y Formación Agraria y Pesquera, Area de Genómica y Biotecnología, Centro de Málaga, Spain

**Keywords:** ascorbic acid, vitamin C, cell wall, biosynthesis, fruit, regulation

## Abstract

Throughout evolution, a number of animals including humans have lost the ability to synthesize ascorbic acid (ascorbate, vitamin C), an essential molecule in the physiology of animals and plants. In addition to its main role as an antioxidant and cofactor in redox reactions, recent reports have shown an important role of ascorbate in the activation of epigenetic mechanisms controlling cell differentiation, dysregulation of which can lead to the development of certain types of cancer. Although fruits and vegetables constitute the main source of ascorbate in the human diet, rising its content has not been a major breeding goal, despite the large inter- and intraspecific variation in ascorbate content in fruit crops. Nowadays, there is an increasing interest to boost ascorbate content, not only to improve fruit quality but also to generate crops with elevated stress tolerance. Several attempts to increase ascorbate in fruits have achieved fairly good results but, in some cases, detrimental effects in fruit development also occur, likely due to the interaction between the biosynthesis of ascorbate and components of the cell wall. Plants synthesize ascorbate *de novo* mainly through the Smirnoff-Wheeler pathway, the dominant pathway in photosynthetic tissues. Two intermediates of the Smirnoff-Wheeler pathway, GDP-D-mannose and GDP-L-galactose, are also precursors of the non-cellulosic components of the plant cell wall. Therefore, a better understanding of ascorbate biosynthesis and regulation is essential for generation of improved fruits without developmental side effects. This is likely to involve a yet unknown tight regulation enabling plant growth and development, without impairing the cell redox state modulated by ascorbate pool. In certain fruits and developmental conditions, an alternative pathway from D-galacturonate might be also relevant. We here review the regulation of ascorbate synthesis, its close connection with the cell wall, as well as different strategies to increase its content in plants, with a special focus on fruits.

## Multiple Roles of Vitamin C in Humans

L-Ascorbic Acid (L-threo-hex-2-enono-1,4-lactone, ascorbate), also called vitamin C, is an essential antioxidant molecule in plant and animal metabolism and also functioning as a cofactor in many enzymes. While many animals are able to synthesize ascorbate in the liver or in the kidney, others, such as humans, non-human primates, guinea pigs, and certain groups of bats and birds have lost this ability due to the accumulation of mutations in the coding sequence of the last committed enzyme of the pathway (L-gulono-1,4-lactone oxidase, GULO; Chatterjee, 1973; Nishikimi et al., 1994; Drouin et al., 2011). Dietary changes with the inclusion of abundant fruits and vegetables in the diet resulted in the loss of selective pressure to keep the pathway functional (Macknight et al., 2017). Thus, this molecule must be incorporated in the diet (hence classified as a vitamin), with vegetables and fruits as the major sources of ascorbate.

The role of ascorbate in mammals has extensively been studied throughout time, particularly since the 18th century with the discovery of its role in preventing scurvy (Lind, 1753; Baron, 2009). However, this was not obvious at the time because the lack of ascorbate in the diet takes about a month before the symptoms to occur. Thus, this disease was typically manifested during long sea travels with a diet scarce in fresh vegetables and fruits. In the earlies 1930’s, Albert Szent-Györgyi identified and isolated the molecule responsible for this anti-scurvy activity. Thus, that molecule, previously called hexuronic acid, was renamed as ascorbic acid. One of the main symptoms in scurvy is skin impairment and injuries due to the involvement of ascorbate in the biosynthesis and stability of collagen. Ascorbate functions as a cofactor in the enzymatic hydroxylation catalyzed by Fe^2+^/αKG-dependent dioxygenases prolyl 4-hydroxylase, prolyl 3-hydroxylase and lysyl hydroxylase (Myllylä et al., 1984; Peterkofsky, 1991; Pekkala et al., 2003; Padayatty and Levine, 2016) through the reduction of Fe^3+^ to the active Fe^2+^ (de Jong et al., 1982; Gorres and Raines, 2010). Prolyl hydroxylation is an essential post-translational modification that occurs in proline residues located at X and Y sites of procollagen Gly-X-Y tandem repeats during collagen biosynthesis. Whereas Prolyl 4-hydroxylases catalyze hydroxylation on Y locations, Prolyl 3-hydroxylases hydroxylate residues located at X sites, thus enabling the trimerization of collagen providing high thermal stability (Koide and Nagata, 2005). The hydroxylation catalyzed by these enzymes requires an Fe^2+^ ion located at the active center, which is oxidized to Fe^3+^ in the catalytic cycle and ascorbate is responsible of keeping the iron active by reducing it back to Fe^2+^.

In addition to preventing scurvy, ascorbate is involved in many other processes which also require the action of other members of this family of mono- and dioxygenases. For these enzymes, ascorbate functions as a cofactor, maintaining activity of the metal ions located in the active centers. For example, ascorbate is important for the synthesis of carnitine, the lack of which is related to the common fatigue found in scorbutic patients. Trimethyllysine hydroxylase and γ-butyrobetaine hydroxylase require ascorbate to enhance their activity in the biosynthesis of carnitine (Rebouche, 1991). In addition, ascorbate is also known to act as a cofactor of dopamine β-monooxygenase (Rush and Geffen, 1980), and in peptide hormone metabolism, by acting as a cofactor of peptidylglycine α-amidating monooxygenase, involved in the C-terminal amidation of these regulatory molecules (Prigge et al., 1999). More recently, the activity of other key Fe^2+^/αKG-dependent dioxygenases have been showed to be enhanced by ascorbate, as is the case of Ten-Eleven Translocations (TETs) enzymes. TETs are involved in DNA demethylation through an oxidation cascade from 5-methylcytosine to 5-hydroxymethylcytosine, 5-formylcytosine, 5-carboxylcytosine and, then, to cytosine by the Base Excision Repair (BER) mechanism (Blaschke et al., 2013; Minor et al., 2013; Hu et al., 2015). Importantly, ascorbate functions as a cofactor of histone demethylases harboring a Jumonji C (JmcC) domain (JHDMs), the same catalytic domain present in TETs (Young et al., 2015). Tri-, di- and monomethylated lysines in histones can be oxidized to hydroxymethyl lysines by JHDM and ascorbate in a similar way as occurring with DNA demethylation and TETs, with an spontaneous removal of this hydroxymethyl group (Young et al., 2015). All together, these findings show that ascorbate participates in the response to environmental stimuli, not only by buffering the cell redox state, but also by its involvement in the epigenetic control on gene expression. In addition, ascorbate enhances iron absorption (Hallberg et al., 1987, 1989), which is not only important to keep the Fe^2+^/αKG-dependent dioxygenases active, but also for many other roles (Lieu et al., 2001; Muckenthaler et al., 2008).

## Major Fruit Supplies of Ascorbate in Humans

Fresh fruits and vegetables are the major sources of this vitamin, therefore increasing its concentration will have an important impact in human nutrition. Ascorbate deficiency in developed countries has registered a decrease throughout time. At the end of last century. ascorbate deficiency in United States was around 13% of the population (Hampl et al., 2004), but it dropped to 7% in the last survey effectuated during 2003-2004 period (Schleicher et al., 2009). According to early experiments, a daily dose of less than 10 mg was found to prevent scurvy (Johnstone et al., 1946; Peters et al., 1953; Baker et al., 1969, 1971; Hodges et al., 1969, 1971). However, an Average Requirement (AR) of 90 mg/day for men and 80 mg/day for women, and a Population Reference Intake (PRI) of 110 mg/day for men and 95 mg/day for women, has been established by the European Food Safety Authority (EFSA Panel on Dietetic Products and Nutrition and Allergies [NDA], 2013). This is based on maintaining a plasma concentration around 50 μmol/L of ascorbate, indicative of an adequate status (Kallner et al., 1979). In United States and Canada, the Recommended Dietary Allowance (RDA) is 90 mg/day for men and 75 mg/day for women (Food and Nutrition Board Panel on Dietary Antioxidants and Related Compounds, 2000).

It is accepted that a diet rich in ascorbate has various health advantages (Wintergerst et al., 2006; Reczek and Chandel, 2015; Carr and Maggini, 2017; van Gorkom et al., 2018). Furthermore, in the last few years, ascorbate has been proposed as a treatment against different types of cancer through various mechanisms, such as increasing TET’s activity, inducing oxidative stress in cancer cells or enhancing the activity of various chemical treatments (Ko et al., 2015; Yun et al., 2015; Agathocleous et al., 2017; Cimmino et al., 2017; Shenoy et al., 2017; Lu et al., 2018; Miura et al., 2018). Daily intake of ascorbate provided by fruits is dependent on several factors, but clearly the content of ascorbate as well as the amount that is consumed are the most important factors. However, it is important to take into account the way it is consumed as this might have important consequences on ascorbate reduction and oxidation, and can also alter the bioavailability of ascorbate due to interactions with other phytochemicals such as Vitamin E or flavonoids (Packer et al., 1979; Tanaka et al., 1997; Carr and Vissers, 2013).

**FIGURE 1:**
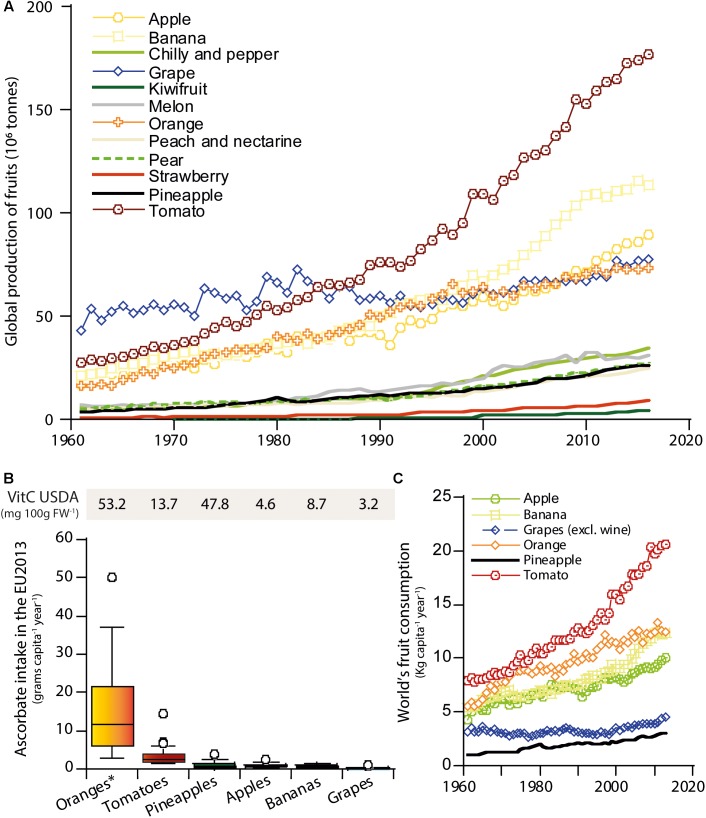
Main fruit crops yield and consumption according to FAO. **(A)** Global fruit production, in million tons, and its evolution from 1961 to 2016. **(B)** Fruit ascorbate intake, in grams of ascorbate capita^-1^ year^-1^, in the countries from the European Union in 2013. Data were generated considering ascorbate (VitC) levels of raw fruit available in USDA database (https://ndb.nal.usda.gov/ndb/search/list) and consumption data of each fruit (Kg capita^-1^ year^-1^) from FAOSTAT. USDA IDs consulted: 9200 (Oranges ^∗^includes mandarins, raw, all commercial varieties), 11529 (Tomatoes, red, ripe, raw, year average), 9003 (Apples, raw with skin), 9132 [Grapes, red or green (European type, such as Thompson seedless), raw], 9266 (Pineapple, raw, all varieties), 9040 (Bananas, raw). Consumption data was obtained from Eurostat (http://ec.europa.eu/eurostat). **(C)** Evolution in the global consumption of fruits, in Kg capita^-1^ year^-1^, from 1961 to 2013.

Ascorbate overall intake is dependent on the intrinsic amount of ascorbate of a specific fruit and its consumption (Figure 1B). According to FAOSTAT^1^, tomato has been the most produced fruit in the world in the last 20 years, a trend that has increased during the last years (Figure 1A). The production has been 177 million tons in 2016, followed by banana (∼113 million tons), apple (∼89 million tons), cucumber (∼80 million tons) and grape (∼77 million tons). In the European Union in 2016, fruit production was dominated by grape (∼24 million tons), followed by tomato (∼18 million tons), apple (∼12.5 million tons) and orange (∼6.3 million tons) (Eurostat, 2017). However, a large proportion of tomato (61.5%), apple (26.8%) and grape (96.5%) is processed (Eurostat, 2017), leading both to a reduction of ascorbate content and a lower bioavailability of other nutrients that are ascorbate dependent (Hallberg et al., 1982, 1987). This is particularly evident in grape, with ∼90 % of the harvest destined to wine production (Eurostat, 2017), leading to negligible amounts of ascorbate (USDA Food Composition Databases^2^). Therefore, considering production along with consumption data (Figure 1C), ascorbate intake through orange surpasses that of grape. Tomato and apple fruits, although could be considered as moderate sources of ascorbate based on their relatively low content (Davey et al., 2000) are widely consumed and therefore provide important dietary sources of ascorbate. It is obvious that even a moderate increase in the content of ascorbate in these highly consumed fruits would rise their nutritional value. Therefore, the large consumption of tomato, its relatively low ascorbate and its high raw intake makes it an excellent target for increasing its ascorbate content from a nutritional point of view (Figure 1).

## The Role of Ascorbate in Plants and Fruits

Ascorbate plays a plethora of roles in plant cells. Important properties of ascorbate are its antioxidant capacity and the finalization of oxidative chain reactions resulting in non-oxidative products such as dehydroascorbate (DHA) and 2,3-diketogulonic acid (Davey et al., 2000). The importance of ascorbate in scavenging ROS became evident when several of the genes involved in the ascorbate biosynthetic pathway were identified in genetic screenings searching for mutants hypersensitive to ozone, a powerful oxidant (Conklin et al., 1996). This screening resulted in the identification of five vitamin C-deficient (*vtc*) mutants, with four of those mutations affecting genes encoding enzymes of the Smirnoff-Wheeler pathway: *VTC1* (Lukowitz et al., 2001), *VTC2* and *VTC5* (Dowdle et al., 2007; Linster et al., 2007) and *VTC4* (Conklin et al., 2006).

Hydrogen peroxide (H_2_O_2_) plays essential roles in plants development and defense (Exposito-Rodriguez et al., 2017; Mittler, 2017; Mullineaux et al., 2018; Waszczak et al., 2018) and it can be found in different organelles within the plant cells (Exposito-rodriguez et al., 2013). However, H_2_O_2_ is also partly responsible for light-induced oxidative damage. Ascorbate is involved in the scavenging of the excess of H_2_O_2_ produced during the photosynthesis in high-irradiance conditions by the function of ascorbate peroxidases (APX), enzymes not present in animals (Wheeler et al., 2015). Together with APX, catalases also perform H_2_O_2_ scavenging (Mhamdi et al., 2010, 2012). However, plants lack catalases in chloroplasts, which experience a high production of H_2_O_2_ in thylakoids due to photosynthesis, as a consequence of the Mehler reaction. In these organelles, a thylakoidal APX (tAPX) catalyzes the reduction of H_2_O_2_ (Asada, 1999). Surprisingly, single and double mutants in chloroplastic APX (tAPX and stromal APX) are viable, suggesting alternative mechanisms for H_2_O_2_ detoxification (Giacomelli et al., 2007). 2-Cys peroxiredoxins (2-Cys PRX), localized in the chloroplast, reduce H_2_O_2_ and prevent oxidation of the thylakoidal membrane by reducing lipid hydroperoxide from thylakoid phospholipids (Baier and Dietz, 1997). Therefore, 2-Cys PRXs have been proposed as alternative H_2_O_2_ scavengers to APX in an alternative water-water cycle (Awad et al., 2015; Pérez-Ruiz et al., 2017) using glutathione, thioredoxin, glutaredoxin, cyclophilin, and/or tryparedoxin instead of ascorbate as cofactors (Stork et al., 2005). Together with APX and 2-Cys PRX, vitamin E (α-tocopherol) is a major lipophilic antioxidant also involved in preventing photodamage in the membrane of thylakoid lipids (Semchuk et al., 2009). Ascorbate also has a role in vitamin E function by the non-enzymatical reduction of α-tocopheryl radicals, hydroxyl radicals (⋅OH) and superoxide ions (O_2_^-^) (Asada, 1999; Davey et al., 2000; Mittler, 2017).

The use of ascorbate as a cofactor by other enzymes, such as the Fe^2+^/α-KG-dependent dioxygenases and Cu^+^-monooxygenases, is conserved among plants and animals. However, one of these common enzymes, a Fe^2+^-dependent 4-hydroxyphenylpyruvate dioxygenase, has different functions in plants. Whereas in animals this enzyme is involved in tyrosine metabolism (Lindblad et al., 1970), in plants it is required for plastoquinone and tocopherols synthesis (Norris et al., 1998). Other light-responsive pigments that are very abundant in fruits, like anthocyanins, fail to accumulate in *vtc1* and *vtc2* mutant plants when exposed to high light. This finding, combined with the UV-B absorption by anthocyanin, suggests that ascorbate-mediated redox reactions act upstream of anthocyanin synthesis (Page et al., 2012).

Ascorbate was proposed to directly participate in photosynthesis as an electron carrier, although later a role as a photoprotectant was revealed (Smirnoff, 2000). The electron transfer from ascorbate to the primary oxidizing agent of photolysis was first coupled to the photophosphorylation reaction (Marrè et al., 1959). Then, the reduction of monodehydroascorbate (MDA) and DHA were suggested to rely on reductants formed in photosystem I (PSI). It is now established that inside the thylakoid, luminal ascorbate acts as an electron donor of photosystem II (PSII) (Tóth et al., 2013) where the Oxygen-Evolving Complex is impaired (Katoh and San Pietro, 1967; Mano et al., 1997; Tóth et al., 2009), thus allowing the reduction of NADP^+^ to NADPH by the electron-transport chain (Tóth et al., 2009, 2013). This is particularly important during abiotic stresses such as heat and high light that alter this complex by damaging the manganese cluster (Tyystjärvi, 2008). In addition, ascorbate can also dissipate energy from an excess of light irradiance acting as a cofactor of violaxanthin de-epoxidase, an enzyme involved in preventing photodamage by non-photochemical quenching (NPQ) (Yamamoto et al., 1972). When the irradiance is too high, the excess of energy normally transferred to chlorophyll *a* is used to de-epoxidize the carotenoid violaxanthin into zeaxanthin using the thylakoid luminal ascorbate as a cofactor in the xanthophyll cycle (Hieber et al., 2000). This has been supported experimentally by mutations in the enzyme’s residues that bind ascorbate (Saga et al., 2010) and by the analysis of Arabidopsis mutants with low ascorbate content (Müller-Moulé et al., 2002).

## Biosynthesis and Metabolism of Ascorbate in Plants

**FIGURE 2:**
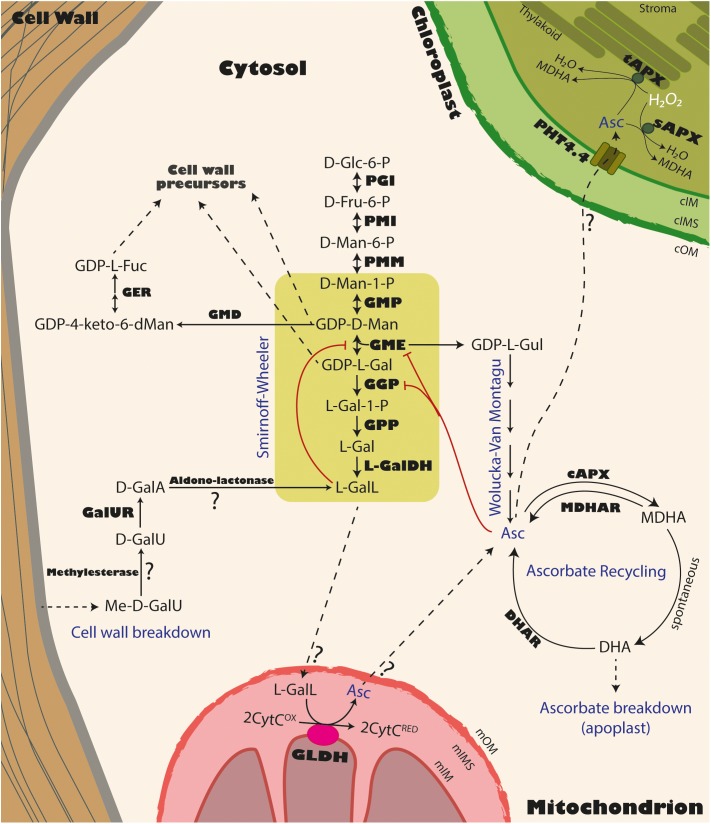
Biosynthesis pathways of ascorbate in the plant cell. Solid lines represent the committed reactions within a pathway. Dashed lines represent the translocation of a molecule from a cellular compartment to another. Enzymes are displayed in bold: PGI, phosphoglucose isomerase; PMI, phosphomannose isomerase; PMM, phosphomannomutase; GMP, GDP-D-mannose pyrophosphorylase (Arabidopsis VTC1); GME, GDP-D-mannose-3′,5′-isomerase; GGP, GDP-L-galactose phosphorylase (Arabidopsis VTC2/VTC5); GPP, L-galactose-1-phosphate phosphatase (Arabidopsis VTC4); L-GalDH, L-galactose dehydrogenase; GLDH, L-galactono-1,4-lactone dehydrogenase; cAPX, cytosolic Ascorbate Peroxidase; MDHAR, monodehydroascorbate reductase; DHAR, dehydroascorbate reductase; PHT4.4, inorganic phosphate transporter; sAPX, stromal ascorbate peroxidase; tAPX, thylakoidal ascorbate peroxidase; GMD, GDP-D-mannose-4,6-dehydratase (Arabidopsis MUR1/GMD1); GER, GDP-4-keto-6-deoxymannose-3,5-epimerase-4-reductase (Arabidopsis GER1/GER2); GalUR, D-Galacturonate Reductase. Substrates and products are shown in regular shape: Glc, glucose; Fru, fructose; Man, mannose; Gal, galactose; Gul, gulose; GalU, Galacturonate; Me-D-GalU, methyl galacturonate; GalA, Galactonate; GalL, L-galactono-1,4-lactone; Asc, ascorbate; CytC^OX^, oxidized cytochrome c; CytC^RED^, reduced cytochrome c; MDHA, monodehydroascorbate; DHA, dehydroascorbate; GDP-α-keto-6-dMan, GDP-4-keto-6-deoxymannose; Fuc, fucose; mOM, mitochondrial outer membrane; mIMS, mitochondrial inter membrane space; mIM, mitochondrial inner membrane; cOM, chloroplastic outer membrane; cIMS, chloroplastic inter membrane space; cIM, chloroplastic inner membrane.

The predominant pathway through which ascorbate is synthesized in plants is the Smirnoff-Wheeler (SW) pathway (Wheeler et al., 1998). Contrary to the animal pathway, in the plant pathway there is no carbon inversion, as the carbon 1 in the D-glucose molecule remains as carbon 1 in ascorbate after conversion. In this pathway, a molecule of D-glucose-6-phosphate is transformed into D-fructose-6-phosphate by the action of phosphoglucose isomerase (PGI; Figure 2). Then, it is transformed into D-mannose-6-phosphate and D-mannose-1-phosphate by the action of phosphomannose isomerase (PMI; Maruta et al., 2008) and phosphomannomutase (PMM; Qian et al., 2007). Then, GDP-D-mannose pyrophosphorylase (GMP, encoded by *VTC1* in *Arabidopsis thaliana*) transfers guanosine monophosphate from GTP to form GDP-D-mannose (Conklin et al., 1996, 1997, 1999; Lukowitz et al., 2001). GDP-D-mannose is further transformed into GDP-L-galactose by the GDP-D-mannose-3′,5′-epimerase (GME), an enzyme that belongs to the extended short chain dehydratase/reductase (SDR) protein family, harboring a modified NAD^+^ binding Rossman fold domain. Interestingly, GDP-L-galactose is not the only result of GME activity, since GDP-L-gulose can also be produced if GME catalyzes a 5′ epimerization instead of a 3′,5′ epimerization (Wolucka et al., 2001; Wolucka and Van Montagu, 2003; Major et al., 2005). Since GDP-L-gulose is a very rare sugar in plants with no structural function, it has been suggested that it is directly channeled to synthesize ascorbate. After GME releases GDP-L-galactose, this compound is then transformed into L-galactose-1-phosphate, L-galactose and L-galactono-1,4-lactone by GDP-L-galactose-phosphorylase (GGP, encoded by *VTC2* and *VTC5* in *A. thaliana*; Dowdle et al., 2007; Laing et al., 2007), L-galactose-1-phosphate phosphatase (GPP, encoded by *VTC4* in *A. thaliana*; Laing et al., 2004a; Conklin et al., 2006; Torabinejad et al., 2009; Nourbakhsh et al., 2015) and L-galactose dehydrogenase (L-GalDH; Gatzek et al., 2002; Laing et al., 2004b), respectively. Interestingly, for the final production of L-ascorbic acid, L-galactono-1,4-lactone must move from the cytosol to the intermembrane space of the mitochondria, where the active site of L-galactono-1,4-lactone dehydrogenase (GLDH) is located (Mapson and Breslow, 1958; Imai et al., 1998; Pineau et al., 2008; Schertl et al., 2012; Schimmeyer et al., 2016). The fact that the oxidation of L-galactono-1,4-lactone is carried out in plants by a dehydrogenase instead of an oxidase (GULO) as occurs in animals, is not trivial. Contrary to paradoxical GULO activity, GLDH does not release H_2_O_2_ and therefore the production of ascorbate in plants does not have side effects over the redox state of the cell (Wheeler et al., 2015). Although some data support the existence of a side branch of the pathway that converges with that of animals (Jain and Nessler, 2000; Radzio et al., 2003; Maruta et al., 2010), there is strong evidence that most of the ascorbate in plants is produced through GLDH (Pineau et al., 2008). A recent phylogenetic study on the origin of GLDH identified an ancient paralog arisen from the original GULO, followed by a loss of paralogs (Wheeler et al., 2015). Thus, in species with the SW pathway, GULO has been functionally replaced by GLDH following chloroplast acquisition in photosynthetic organisms, since the presence of both proteins seems mutually exclusive (Wheeler et al., 2015). Interestingly, L-gulose, a previously mentioned rare sugar in plants and also a product of GME activity, is proposed to be transformed into L-gulono-1,4-lactone by as yet unidentified enzymes (Wolucka and Van Montagu, 2003). Supporting the presence of GULO activity in plants are (1) external supplementation of L-gulono-1,4-lactone in the growth media increased ascorbate levels in WT tobacco leaves (Jain and Nessler, 2000) and (2) the synthesis rate of ascorbate can increase up to 15% when L-gulono-1,4-lactone is externally supplied in Arabidopsis cell culture (Davey et al., 1999). One possibility is that GLDH also uses L-gulono-1,4-lactone as substrate. However, this seems unlikely since GLDH is highly specific for L-galactono-1,4-lactone (Mapson and Breslow, 1958; Oba et al., 1995; Østergaard et al., 1997; Rodríguez-Ruiz et al., 2017). Transgenic tobacco BY2 cells overexpressing several Arabidopsis homologs of GULO from rat resulted in increased ascorbate content in lines overexpressing GULO2, GULO3 and GULO5 but only after external application of L-gulono-1,4-lactone (Maruta et al., 2010). However, GULO has lower substrate specificity than GLDH and can catalyze the oxidation of other aldono-lactones, including L-galactono-1,4-lactone (Davey et al., 2000). Interestingly, the overexpression of rat liver GULO increased ascorbate levels in tobacco leaves (Jain and Nessler, 2000) as well as in Arabidopsis leaves, and rescued the Arabidopsis *vtc1* mutant ascorbate levels to WT (Radzio et al., 2003).

Alternative ascorbate biosynthesis pathways have been proposed in plants. One is through myo-inositol, following a pathway similar to animals, since the oxidation of myo-inositol oxidation produces D-glucuronate by a *MYO-INOSITOL OXYGENASE* (*MIOX*). Arabidopsis plants overexpressing *MIOX4* showed a 2-3-fold ascorbate content (Lorence et al., 2004). However, based on early radiotracer experiments (Loewus, 1963) and more recent reports (Endres and Tenhaken, 2009, 2011; Ivanov Kavkova et al., 2018), its contribution to the ascorbate pool remains unclear. The second is through the D-galacturonate pathway. In this pathway, a D-galacturonate reductase (GalUR) uses D-galacturonate, to produce L-galactonic acid that is converted to L-galactono-1,4-lactone, the last intermediate within the SW pathway (Mapson and Isherwood, 1956; Shigeoka et al., 1979).

In addition to its biosynthesis, the ascorbate pool also depends on its recycling by the Foyer-Halliwell-Asada cycle (Foyer and Halliwell, 1976; Asada, 1999) and degradation (Loewus, 1999; Green and Fry, 2005). Although the biochemistry of biosynthesis and recycling of ascorbate is well established, its degradation is not clear and might not follow a single pathway. In the apoplast, it can be degraded through the conversion of ascorbate to 2-keto-L-gulonic acid that leads to L-tartaric acid formation in cytoplasm, a compound important for fruit quality particularly in the *Vitaceae* family (DeBolt et al., 2006). Ascorbate can also be degraded through the direct oxidation of DHA or through the oxidation of 4-O-oxalyl-L-threonic acid, leading to the production of both oxalic acid and L-threonic acid (Green and Fry, 2005). Additionally, it can also be degraded through the hydrolysis of DHA to 2,3-diketo-gulonic acid, and to oxalic acid and its esters, or to L-threonic acid under strong oxidative conditions (Parsons et al., 2011). In tomato, the main degradation products are oxalic acid, threonic acid and oxalyl threonic acid, but no tartaric acid has been detected (Truffault et al., 2017), suggesting that ascorbate degradation occurs mainly through DHA oxidation rather than DHA hydrolysis, a pathway previously proposed in *Rosa sp.* cell cultures (Green and Fry, 2005). A broad perspective of ascorbate breakdown pathways in different species is provided by DeBolt et al. (2007).

## Biosynthesis and Metabolism of Ascorbate in Fruits

Mutant analyses indicate that the SW pathway is the predominant if not the only pathway involved in ascorbate biosynthesis in green tissues (Dowdle et al., 2007; Lim B. et al., 2016). In heterotrophic tissues like fruits, the SW pathway is functional, as showed in several species including acerola, kiwi, strawberry, peach, tomato and apple (Badejo et al., 2009, 2012; Bulley et al., 2009; Imai et al., 2009; Ioannidi et al., 2009; Cruz-Rus et al., 2010; Mellidou et al., 2012a,b). However, depending on the fruit ripening stage, alternative pathways might become relevant, especially the D-galacturonate pathway (Mapson and Isherwood, 1956; Shigeoka et al., 1979), for which the degradation of cell wall pectin can provide abundant substrate (Agius et al., 2003; Cruz-Rus et al., 2010; Di Matteo et al., 2010; Badejo et al., 2012). Analyses of tomato introgression lines from a cross between *Solanum lycopersicum cv*. M82 and *S. pennellii* was used to find genetic elements associated with high ascorbate content in fruits. This was done through the identification of genes induced in the IL12-4 line, which contains 19.9 mg ascorbate/g FW, relative to *S. lycopersicum cv.* M82, which contains 12.2 mg ascorbate/g FW (Di Matteo et al., 2010). Interestingly, while genes of the SW pathway were not differentially expressed, a pectinesterase gene (TC177576) involved in breakdown of pectins was 4.4-fold more expressed in the IL12-4 line than in the parental M82. This result suggests that an additional supply of D-galacturonate due to cell wall degradation might be the cause of the ascorbate increase in this line. In addition, an ascorbate peroxidase (TC172881) was down-regulated in fruits of IL12-4 compared to M82 parental line, which may cause a higher ascorbate accumulation due to a lower degradation (Di Matteo et al., 2010). While the D-galacturonate pathway is more active as the fruit ripens, the SW pathway and ascorbate translocation from the leaves provide the bulk of ascorbate in fruits at immature green stage. The fact that the photosynthesis inhibitor DCMU diminished the pool of ascorbate only at green stage (Badejo et al., 2012) not only supports this, but also reinforces the tight relationship between the SW pathway and photosynthesis.

Considering the variety of functions that ascorbate exerts in plant cells and its tight regulation in green tissues, it is remarkable how variable the content of ascorbate can be among the fruits of different species (Davey et al., 2000) or even between varieties or cultivars from the same species (Cruz-Rus et al., 2011; Mellidou et al., 2012b). An obvious question is: what is the functional significance of this high variability in fruit ascorbate content? Fruit crops have different environmental requirements to optimize yield and, in addition, the pool of ascorbate is affected by abiotic factors such as light or temperature (Gautier et al., 2008; Zechmann et al., 2011; Suzuki et al., 2014), due to its role in the antioxidant cellular system (Jimenez et al., 2002; Massot et al., 2013). Therefore, small differences within species can depend on their cultivation requirements, harvest time or post-harvest conditions (Davey et al., 2007; Kevers et al., 2011; Oms-Oliu et al., 2011; Akhatou and Fernández-Recamales, 2014). However, the observed large differences in ascorbate content in closely related species likely have other causes. For example, several fold differences in ascorbate can be found between wild and cultivated tomato. Whereas domesticated tomato cultivars contain roughly 15 mg/100 g FW, wild varieties *S. pimpinellifolium* and *S. pennellii* contain around 40 mg/100 g FW (Lima-Silva et al., 2012) and up to 70 mg/100 g FW (Stevens et al., 2007), respectively. In fact, back-crosses with *S. peruvianum*, another wild species (Atherton and Rudich, 1986), have been shown to contain the highest amount of ascorbate in *Solanum* species, around 50 mg/100 g FW (Top et al., 2014). These wild tomato species grow naturally in Peru and Mexico, in coastal areas and river valleys less than 1000 m above sea level with abundant rainfalls. These two countries lie within the tropics of Capricorn and Cancer, respectively, with high irradiance and warm temperatures that may have favored the selection of individuals with high ascorbate content over time. Current evidence suggests that domestication of wild tomatoes by cross-breeding different species of *Solanum* started in these two countries (Esquinas-Alcazar, 1981) likely driven by the selection of higher fruit size and resistance to diseases like *Fusarium* wilt (Atherton and Rudich, 1986). However, the most important advances in tomato breeding have taken place during the last 200 years in Europe, mainly in France, Italy and England, with a strong participation of the United States since the early 1920s (Atherton and Rudich, 1986). It is likely that growing under more controlled and less harsh conditions has decreased the selective pressure to keep alleles conferring high ascorbate content, particularly because an apparent association between high ascorbate levels and low productivity has been reported in this species (Atherton and Rudich, 1986). However, in addition to the metabolic regulatory mechanisms that might explain these differences in ascorbate content, other factors such as water content must be considered. A known example is that of two tomato cultivars, Matador and Elin, subjected to salinity treatment. This increased their ascorbate content on fresh weight basis, but it was decreased on dry weight basis. In both cultivars, water and ascorbate content were reduced, but the loss of water was higher than that of ascorbate (Dumas et al., 2003). Fruit size and weight were directly related with water content, and they have been key traits selected during breeding programs, while this is not the case for ascorbate.

In most fruits, such as tomato, acidity decreases while sugar content increases during ripening (Gautier et al., 2008; Mellidou et al., 2012b). Major organic acids in tomato, contributing to fruit acidity, are malic and citric acids (Davies and Hobson, 1981). However, the change in ascorbate levels during fruit ripening is a trait dependent on the species. In tomato (Dumas et al., 2003; Gautier et al., 2008; Ioannidi et al., 2009; Badejo et al., 2012), grape (Cruz-Rus et al., 2010) and strawberry (Cruz-Rus et al., 2011), ascorbate content increases as the fruit ripens. This correlated with changes in the activity of enzymes affecting the redox state of the fruit during the breaker stage (Gautier et al., 2008; Jimenez et al., 2002). Unlike tomato, grape and strawberry, kiwifruit showed a maximal ascorbate level at the immature green stage due to its high biosynthesis rate, it decreased as it ripened and then remained fairly stable until complete ripening (Li et al., 2010; Zhang J.Y. et al., 2018). In peach fruits, ascorbate content gradually decreased during ripening (Imai et al., 2009). In different studies, the pattern of ascorbate accumulation does not match the expression of a specific gene involved in ascorbate biosynthesis or recycling, and therefore there is no clear connection between the expression of biosynthetic genes and ascorbate content (Imai et al., 2009; Li et al., 2010; Lima-Silva et al., 2012). However, evidences gathered in these studies show that the overall size of the ascorbate pool correlated well with the oxidative status (i.e., activity of enzymes involved in redox state, H_2_O_2_ content) of the fruit, which is usually triggered at breaker stage (Jimenez et al., 2002; Gautier et al., 2008; Imai et al., 2009; Li et al., 2010).

## Relationship Between Ascorbate and Cell Wall Biosynthesis

**FIGURE 3:**
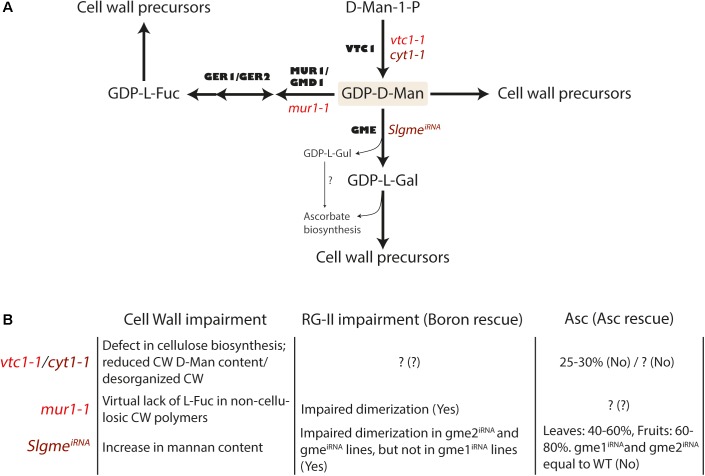
GDP-D-mannose and its biological relevance for ascorbate and cell wall biosynthesis in plants. **(A)** Reaction scheme for the novo synthesis of GDP-D-mannose in *Arabidopsis thaliana*. Mutants described for each step are indicated in lower case italic red letters. **(B)** Biological impairment over cell wall (RG-II, rhamnogalacturonate II) and ascorbate content in mutants of genes controlling the GDP-D-mannose pool. MUR1 and GMD1 encode two GDP-D-mannose-4,6-dehydratases. GER1 and GER2 encode two GDP-4-keto-6-deoxymannose-3,5-epimerase-4-reductases. The epimerase reaction is reversible whereas the reduction is not (Bonin et al., 1997). VTC1 encodes GMP, a GDP-D-mannose pyrophosphorylase, GME encodes a GDP-D-mannose-3′,5′-isomerase. D-Man-1-P, D-mannose-1-phosphate; GDP-D-Man, GDP-D-mannose; GDP-L-Gul, GDP-D-gulose; GDP-D-Gal, GDP-D-galactose; GDP-D-Fuc, GDP-D-fucose; Asc, Ascorbate.

A significant aspect of the ascorbate biosynthetic pathway is the intimate relationship shared with cell wall biosynthesis. Some of the early precursors of the SW pathway such GDP-D-mannose and GDP-L-galactose are among the non-cellulosic cell wall glycosyl residues forming pectins and hemicelluloses (Figure 3). For this reason, mutations or knock-downs in genes related to the early steps of the SW pathway lead to growth reduction or even arrest, due to impairment of cell wall formation during plant growth, including different stages of fruit development (Lukowitz et al., 2001; Hoeberichts et al., 2008; Mounet-Gilbert et al., 2016). Thus, knock-down mutants of the Arabidopsis *PMM* gene show between 20 and 50% of ascorbate levels relative to WT, altered protein N-glycosylation (specially a protein-disulfide isomerase post-translational modification, an abundant protein in the ER) and glycosylphosphatidylinositol (GPI) anchoring of proteins, leading to cell death after heat stress (Hoeberichts et al., 2008). Supplementation with L-galactono-1,4-lactone (Hoeberichts et al., 2008) or ascorbate (Cho et al., 2016) in the media recovered ascorbate levels but the mutants remained hypersensitive to heat. A null mutation in the Arabidopsis *GMP* gene (*cyt1* mutant) results in embryo arrest due to defects in *N-*glycosylation of proteins and altered composition of the cell wall (Figure 3; Lukowitz et al., 2001). The product of GMP activity, GDP-D-mannose, is used in the glycosylation of proteins, ascorbate biosynthesis and as a precursor of cell wall carbohydrates (Conklin et al., 1999). GDP-D-mannose is converted to GDP-L-galactose by the action of GME, but can also be converted to GDP-L-fucose by the sequential function of GDP-D-mannose-4,6-dehydratase (MUR1/GMD1; Bonin et al., 1997, 2003) and GDP-4-keto-6-deoxy-mannose-3,5-epimerase/4-reductase (GER1/GER2; Bonin and Reiter, 2000; Nakayama et al., 2003; Figure 3). All these three compounds, GDP-D-mannose, GDP-L-galactose and GDP-L-fucose are precursors of hemicelluloses and pectins (RG-II) when converted to D-mannosyl, L-galactosyl and L-fucosyl residues (Conklin et al., 1999; Reiter and Vanzin, 2001).

All the above evidences support the conclusion that a reduction in the production of GDP-D-mannose in the *cyt1* mutant is expected to have a significant impact on the structure of the cell wall. The importance of GDP-D-mannose in cell wall structure was further supported by the identification of the *mur1* mutant (Bonin et al., 1997). Mutations in *MUR1* produce a dwarf phenotype, mainly caused by a reduced content in fucose, since the supply of exogenous L-fucose reverted the dwarf phenotype (O’Neill et al., 2001). Interestingly, L-fucosyl residues in *mur1* cell wall xyloglucans are replaced by L-galactosyl residues (Reiter et al., 1993; Zablackis et al., 1996; Bonin et al., 1997). One possibility is that this substitution is the direct cause the dwarf phenotype of *mur1*. However, this does not seem to be the case since the Arabidopsis *mur2* mutant, affected in a xyloglucan-specific fucosyltransferase (*AtFUT1*), grows indistinguishably from WT despite having around 1% of the L-fucose content of the WT (Perrin et al., 1999; Vanzin et al., 2002). Moreover, the xyloglucans of jojoba seeds naturally contain L-galactosyl residues, and not fucosyl residues (Hantus et al., 1997; Pauly and Keegstra, 2016), suggesting that xyloglucan substitution of L-fucose by L-galactose residues is not the cause of growth impairment in *mur1*. In addition to this replacement of fucosyl by L-galactosyl residues in xyloglucan, the *mur1* mutant also has the same substitution in their RG-II fraction of pectins. In the RG-II structure, cross-linking mediated by boron is essential for a proper dimerization (O’Neill et al., 2001). Therefore, an alternative possibility was that changes in monosaccharide composition in the pectic RG-II *mur1* can impair this dimerization, which in turn would lead to dwarfism. In fact, the impaired dimerization in RG-II seems to be the cause of this dwarf phenotype because exogenous application of boron restituted the wild type phenotype (O’Neill et al., 2001). This is consistent with the finding that *mur2* mutants are neither affected in RG-II cross-linking nor L-fucose content (O’Neill et al., 2001). Furthermore, Arabidopsis *cgl* mutants, lacking the N-acetyl glucosaminyl transferase I in their Golgi apparatus (and hence, L-fucosylation), do not present altered growth (von Schaewen et al., 1993). Altogether, growth defects in *mur1* point to a structural defect which is due to impairment in RG-II dimerization, and not due to defects in protein fucosylation. However, defective interactions with different cell wall polymers cannot be completely ruled out, since the α-1,3-xylosyltransferase activity carried out by RGXT enzyme family, involved in RG-II synthesis, transfers D-xylose residues from UDP-xylose onto fucose (Egelund et al., 2006).

An additional link between ascorbate and cell wall biosynthesis comes from studies of tomato lines silencing *GME* (Gilbert et al., 2009; Mounet-Gilbert et al., 2016). Those lines with both copies of *GME* silenced showed reduced growth, higher fragility, lower fruit firmness and a 35–55% reduced ascorbate content in leaves and 20–40% of WT ascorbate levels in fruits (Gilbert et al., 2009). Consistent with the expected accumulation of GDP-D-mannose, the silenced lines showed an increase in mannose-linked cell wall and defects in dimerization of RG-II by boron-mediated cross-linking, since phenotypic defects could be rescued by the application of external boron, but not with ascorbate (Gilbert et al., 2009; Voxeur et al., 2011; Mounet-Gilbert et al., 2016; Qi et al., 2017). All these results strongly suggest that this impairment has a cell wall structural basis rather than reduced ascorbate levels, similar to what was previously found in an Arabidopsis *mur1-1* mutant (O’Neill et al., 2001). Supporting this connection between ascorbate and cell wall biosynthesis at the GDP-D-mannose level, inactivation of GMP activity by knocking down Arabidopsis *KONJAC* genes involved in the activation of GMP resulted in reduced glucomannan content of cell walls and severe dwarfism (Sawake et al., 2015). The overexpression of *KONJAC1* caused a slight increase in ascorbate, whereas it resulted in a significant increase in the glucomannan content of plant cell walls, suggesting the presence of a mechanism that limits ascorbate accumulation.

This interaction between cell wall and ascorbate biosynthesis does not rely only on sharing common intermediates. As an enzyme cofactor, ascorbate is required for the activities of proline and lysine hydroxylases that, as previously mentioned, are involved in collagen biosynthesis in animals. In plants, proline hydroxylation is required for the production of hydroxyproline-rich glycoproteins (HRGP) such as arabinogalactans (AGPs) and extensins (EXTs). These proteins are part of cell wall structural glycoproteome acting as scaffolding components (Kishor et al., 2015; Marzol et al., 2018). AGPs are highly glycosylated HRGP proposed to function as cross-linkers of different cell wall polymers, thus conferring plasticity to the cell wall (Lamport et al., 2006). Recently, AGPs have been shown to be structural components of the cell wall by covalent attachment to pectins (rhamnogalacturonan I/homogalacturonate) and hemicelluloses (arabinoxylan), giving rise to the Arabinoxylan Pectin Arabinogalactan Protein complex APAP1 (Tan et al., 2013). In plants, EXTs have a role similar to that played by collagen in animals but contrary to collagen, EXTs can undergo *O*-glycosylation. This post-translational modification leads to oligo-arabinosylation of hydroxyproline residues that allow the formation of a three-dimensional network *in muro*, attaching to other cell wall components such as pectins (Hijazi et al., 2014; Kishor et al., 2015). Indeed, proline hydroxylation is the preceding step to *O*-glycosylation of extensins and arabinogalactans (Showalter and Basu, 2016). Overall, proline hydroxylase activity, promoted by ascorbate, is essential for cell wall assembly and stiffening. Conversely, ascorbate has been implicated in fruit softening through non-enzymatic mechanisms, mainly by solubilizing pectins due to •OH radicals arising as a result of the Fenton reaction in the apoplast (Dumville and Fry, 2003). Because the architecture of pectins in the seed coat is important in interactions with other cell wall polymers (Turbant et al., 2016), this ascorbate-driven decrease in pectins might lead to seed abortion. These seemingly opposite effects of ascorbate in the cell wall can be explained by a fine-tuned regulation of the ascorbate content and its compartmentalization, aspects that are still poorly understood.

## Regulation of Ascorbate Content

As an essential antioxidant, regulation of the ascorbate content is closely related with abiotic stresses that normally cause oxidative stress. High light in particular is translated into a ROS burst caused by an increased photoreduction and photorespiration. This, in turn, leads to increased ascorbate biosynthesis in order to detoxify these ROS (Asada, 1999). Low light, in contrast, causes a reduction of ascorbate. For example, *Arabidopsis* plants grown in continuous dark for 2 days only contained 20% of ascorbate relative to plants grown in light (Conklin et al., 2013).

Regulatory mechanisms that control ascorbate biosynthesis have been found at the level of transcription, translation, protein stability and activity for different components of the SW pathway. Light modulation of ascorbate content involves GMP stability (Wang et al., 2013), since GMP protein is degraded in the dark by the CONSTITUTIVE PHOTOMORPHOGENIC9-Signalosome subunit 5B (*CSN5B*; Wang et al., 2013). At the transcriptional level, low light decreases the expression of *GGP*, whereas high light causes its induction (Dowdle et al., 2007). Similarly, high light also induces the expression of *GLDH* in melon (Pateraki et al., 2004). NO treatment, which induces oxidative stress, causes an increase of *GLDH* mRNA levels in pepper (Rodríguez-Ruiz et al., 2017). At the activity level, *Arabidopsis* and barley plants exposed to high light showed an increment of GGP and GLDH activity (Smirnoff, 2000; Dowdle et al., 2007), A redox regulation has also been reported for the activities of L-GalDH in kiwifruit (Laing et al., 2004b), GME (Wolucka and Van Montagu, 2003) and GLDH (Leferink et al., 2009) in Arabidopsis. For GLDH in particular, Cys-340 has been identified as a redox-sensitive thiol residue required for an optimal conversion of L-galactono-1,4-lactone into ascorbate. This residue can be irreversibly oxidized by H_2_O_2_ unless it is previously S-glutathionylated (Leferink et al., 2009). This oxidation might be involved in the programmed cell death induced by some stresses like heat, since GLDH activity decreases during early stages of programmed cell death resulting in the inhibition of ascorbate biosynthesis (de Pinto et al., 2015). Therefore, the increased conversion of L-galactono-1,4-lactone to ascorbate under oxidative stress or high light might be an important control point of ascorbate biosynthesis (Smirnoff, 2000).

Probably, the best described regulatory control point of ascorbate biosynthesis is exerted by GGP (Laing et al., 2015). This study reports that the amount of GPP protein in Arabidopsis is controlled by a *cis*-acting upstream Open Reading Frame (uORF). Thus, at high ascorbate concentration there is a decrease of the translation of *GGP* mRNA, functioning as a negative feedback loop (Laing et al., 2015). More importantly, since this uORF has been identified in *GGP* genes from mosses to angiosperms, this ascorbate post-translational regulation is likely conserved throughout many plant species. Another possible control point exerted by ascorbate is L-GalDH, since the activity of this enzyme purified from spinach leaves is inhibited by ascorbate (Mieda et al., 2004). However, this is now under debate based on activity studies of the purified L-GalDH from kiwifruit (Laing et al., 2004b). The role of GLDH in ascorbate biosynthesis has also been studied during fruit development of tomato and pea. GLDH activity is inhibited by high ascorbate levels (Pallanca and Smirnoff, 2000; Mellidou et al., 2012b), a feedback control also found to affect GME activity in Arabidopsis (Wolucka and Van Montagu, 2003). Another link related with stress came with the finding that the activity of PMM is enhanced by a Ca^2+^-dependent interaction with Calmodulin-Like 10 (CML10; Cho et al., 2016), of which the expression is boosted by H_2_O_2_ and biotic stress (Zimmermann et al., 2004). Accordingly, *Arabidopsis* transgenic lines expressing an artificial microRNA against CML10 fail to increase ascorbate levels under heat stress (Cho et al., 2016).

Other genes involved in the regulation of ascorbate levels are *AMR1* (Zhang et al., 2009) and *ERF98* (Zhang et al., 2012). AMR1 encodes an F-Box protein that represses the expression of virtually all the SW genes, particularly the expression of *GME* and *GGP*. Interestingly, this negative regulator of the pathway is barely expressed under high light conditions, pointing out the importance of the *de novo* biosynthesis of ascorbate in the response to light (Zhang et al., 2009). In contrast, *ERF98* is a positive regulator of the pathway since overexpression of this gene increase the content of ascorbate by enhancing the expression of genes of the SW pathway, in particular *GMP*, *GGP* and *L-GalDH*. Further analysis indicated that ERF98 can directly bind the promoter of the *GMP* gene (Zhang et al., 2012), supporting its regulatory role of the SW pathway.

An important aspect concerning ascorbate regulation is how it is distributed at the subcellular level. Cytohistochemical analysis, based on immunogold labeling and high-resolution immuno electron microscopy in tobacco and Arabidopsis leaves have shown that ascorbate is unevenly distributed at the subcellular levels (Zechmann et al., 2011). The estimated concentrations of ascorbate in Arabidopsis are: mitochondria (10.4 mM), chloroplasts (10.8 mM), peroxisomes (22.8 mM), nuclei (16.3 mM), vacuole (2.3 mM) and cytosol (21.7 mM) (Zechmann et al., 2011). In addition, low concentrations of ascorbate (0.002 mM) and DHA (0.36 mM) have been reported in the apoplast (Booker et al., 2012). These concentrations vary when plants are exposed to high light, which translates into an increase of ascorbate content in most cell compartments (Zechmann, 2011; Zechmann et al., 2011) with the exception of peroxisomes, whose content diminishes under high light. Interestingly, vacuolar ascorbate increases fourfold when plants are exposed high light. This might be necessary to reduce the phenoxyl radicals that are oxidized by the high light associated-increase of H_2_O_2_ (Takahama, 2004). However, it is unknown whether the increase in ascorbate content in vacuole is due to the reduction of vacuolar MDHA through *trans*-membrane ascorbate-mediated electron transporters like cytochrome b561 (Griesen et al., 2004; Asard et al., 2013) or by direct transport of cytosolic ascorbate into the vacuole under high light using a transporter not identified yet.

Interestingly, despite the low concentration of ascorbate, the apoplast ratio of ascorbate/DHA ascorbate is important to determine the redox state of this compartment, which it turn controls redox-dependent signaling processes (Waszczak et al., 2018), such as stomata closure (Chen and Gallie, 2004) and chloroplast reprogramming leading to light acclimation (Karpinska et al., 2018). All these processes would be compromised if DHA and MDHA were not reduced back into ascorbate. Considering the little amount in the apoplast of glutathione and the enzymes in the Halliwell-Asada cycle other mechanisms must keep the redox homeostasis or the apoplast. First, apoplastic DHA produced by the spontaneous oxidation of MDHA enters the cytosol in exchange with ascorbate through facilitated diffusion using a yet-unknown protein (Horemans et al., 1996, 1997, 1998). Once in the cytosol, DHA is reduced to ascorbate by DHAR through the glutathione cycle. Second, MDHA is reduced to ascorbate in the apoplast by a cytochrome b-mediated *trans*-plasma membrane electron transport that uses cytosolic ascorbate as an electron donor (Horemans et al., 1994, 2000), which resembles the ascorbate restoration by electron transport across the tonoplast membrane (Asard et al., 2013), thus suggesting the involvement of cytochrome b561 in the reduction of apoplastic MDHA.

A similar question remains concerning MDHA and DHA reduction back to ascorbate in the thylakoid lumen. Taking into account the importance of luminal ascorbate in the maintenance of the functionality of the photosynthetic apparatus and energy dissipation (NPQ) commented above, MDHA and DHA must be reduced back to ascorbate. Since, to the best of our knowledge, there are no DHA reductases (DHAR) nor MDHA reductases (MDHAR) in the thylakoid lumen, other mechanisms should be involved. It has been shown that luminal DHA, produced by MDHA disproportionation in the lumen, crosses the thylakoidal membrane to the stroma (Mano et al., 1997), where it is reduced by the Halliwell–Asada cycle (Asada, 1999). The mechanism by which DHA crosses the thylakoidal membrane is not clear. Since no DHA transporter has been yet described in thylakoids (Foyer and Lelandais, 1996; Foyer and Noctor, 2011), the difference in DHA concentration between stroma and thylakoid lumen, and the lack of charge, would favor a high diffusion rate toward the stroma. On the other hand, ascorbate (newly synthetized and recycled from DHA) has to enter the lumen of the thylakoid. The diffusion hypothesis might also apply if the concentration of ascorbate in the stroma is much higher than that in the lumen, consistent with a non-active transport of ascorbate into the lumen previously reported (Foyer and Lelandais, 1996). However, unlike DHA, ascorbate has a negative charge making it a less suitable molecule to diffuse across the thylakoid membrane (Horemans et al., 2000). It has been recently reported that AtPHT4;4 transports ascorbate from the chloroplastic intermembrane space into the stroma (Miyaji et al., 2015). Interestingly, the homologous AtPHT4;1 is localized in the thylakoid membrane (Pavón et al., 2008) and its expression is modulated by light (Guo et al., 2008; Miyaji et al., 2015). Therefore, AtPHT4;1 is a good candidate to transport ascorbate across the thylakoid membrane.

## Approaches to Increase Ascorbate in Fruits

Increasing ascorbate content in highly consumed fruits would clearly have an impact on human nutrition. A concomitant increase of ascorbate in tissues or organs that are submitted to oxidative stress, i.e., photosynthetic tissues, might have an additional beneficial effect on plant tolerance. However, whether or not ascorbate increases in fruit would have an effect on stress tolerance is not so clear, although is proposed that during fruit development and ripening oxidative stress might occur (Brennan and Frenkel, 1977; Rogiers et al., 1998; Jimenez et al., 2002; Huan et al., 2016). Most of the attempts used to increase ascorbate levels are based on biotechnology and basically consist in the overexpression of genes involved in different aspects of ascorbate metabolism (biosynthesis, recycling, or regulation). A second approach to increase the content of ascorbate would be through the selection of specific genomic regions that determine high ascorbate from a donor cultivar (or related species) and introgression into the cultivar of interest using molecular-assisted breeding (Singh and Singh, 2015). While in the first approach it is possible to use genes from different species and promoters that drive high or specific expression in desired tissues (Amaya et al., 2015), as far as the target species is amenable of transformation, the second approach relies in the identification of natural variants that can be used to inter-cross with these lines of interest. Although to date there are limited reports using this approach, the clear advantage is that these lines can be directly put into production because it does not involve transgenesis and therefore are not subjected to GMO regulation (Huang et al., 2016).

### Biotechnological Approaches

There are abundant reports in the literature showing an increase of ascorbate in plants using biotechnological approaches (Valpuesta and Botella, 2004; Macknight et al., 2017; Mellidou and Kanellis, 2017). However, most of the studies have been performed in plants that do not produce edible fruits such as Arabidopsis, tobacco or rice and thus, most of the analyses were focused on vegetative tissues. Within fruits, tomato has been the preferred model due to its adoption as a model of fleshy fruits, its commercial value and the availability of efficient transformation protocols. The highest increase of ascorbate in tomato fruits reported so far has been about sixfold and was achieved by ectopically expressing *GGP* from kiwi (Bulley et al., 2012). Interestingly, the transgenic tomato lines with the highest increase of ascorbate showed fruits with developmental defects and did not produce seeds (Bulley et al., 2012). A possible explanation is that an increase of metabolic flux to the synthesis of ascorbate had the effect of draining metabolites that are required for cell wall biosynthesis, particularly during seed development. Alternatively, this sharp increase of ascorbate might cause an increase in pectin solubilization (Dumville and Fry, 2003), which might provoke defects in seed development. Interestingly, in the same study, overexpression of *GGP* also caused an increase in ascorbate content of approximately twofold in strawberry without obvious defects during seed formation. There can be several explanations for these differences in fruit development, first the ascorbate increase in strawberry fruit is smaller, thus not being enough to solubilize pectins, second strawberry is a false fruit with the real fruits (the achenes) located outside the fleshy part, and third the composition of the cell wall surrounding the fruits might be different in terms of pectin composition.

Genes involved in ascorbate biosynthesis from alternative pathways have also been used to increase ascorbate content in tomato fruit. Three different studies in tomato have been published expressing the *D-Galacturonate Reductase* (*GalUR*) gene from strawberry (Agius et al., 2003). In two reports, overexpression of *GalUR* caused an increase between 2 and 2.5-fold, which resulted in enhanced tolerance to various abiotic stresses (Cai et al., 2015; Lim M.Y. et al., 2016). In the third study, *GalUR* is driven by the constitutive 35S promoter or the tomato fruit-specific *polygalacturonase* (*PG*) promoters (Amaya et al., 2015). In both cases, transgenic lines showed a modest (1.3-fold) increase of ascorbate content. However, a comprehensive metabolomic analysis indicated complex changes in metabolites as well as concomitant increase of total antioxidant capacity in transgenic tomato fruits, suggesting that the increase of ascorbate is associated with a tight regulation of the cellular redox state of fruits.

Other approaches have employed genes involved in ascorbate recycling or transcription factors involved in the regulation of genes of the SW pathway. Overexpression of the cytosolic *DHAR1* gene from potato increased the ascorbate content by 1.9-fold in transgenic tomato fruits (Li et al., 2012). Two additional reports using regulatory factors also show a modest increase of ascorbate in fruits. Identification and overexpression of SlHZ24, a transcription factor that binds the promoter of the tomato *SlGMP3* gene (Hu et al., 2016), caused a 1.6-fold increase of total ascorbate in tomato fruits at the breaker stage. Further analysis indicated that SlHZ24 also can bind *in vitro SlGME2* and *SlGGP* promoters, suggesting that this transcription factor can target multiple genes involved in ascorbate biosynthesis. The tomato *SlDof22* negatively regulates ascorbate accumulation in tomato, and reduction of the endogenous expression of this gene by RNAi increased the levels of ascorbate 1.3-1.6-fold in mature fruits. Transcriptomic analysis indicated that the *SlDof22* silenced lines had increased expression of several genes involved in the SW pathway and recycling of ascorbate (Cai et al., 2016). Further, the authors showed that *SlDof22* can bind the promoter of the tomato *SOS1*, a Na^+^/H^+^ antiporter involved in Na^+^ homeostasis and essential for salt tolerance (Zhu, 2002). However, how the *SOS* pathway and the ascorbate biosynthetic pathway are connected remains elusive.

From a breeding perspective, the increases of ascorbate between 1.5 and 2-fold using biotechnological approaches in tomato here reported might not seem outstanding (Amaya et al., 2015; Cai et al., 2015; Lim M.Y. et al., 2016). However, considering the large consumption of tomato, its relatively low ascorbate and its high raw intake, we believe that the reported increments would have a positive impact from a nutritional point of view, more so considering the recent reports on the health beneficial effects of a rich ascorbate diet. The sixfold ascorbate increase reported by Bulley and coworkers (Bulley et al., 2012) would have a tremendous impact on ascorbate intake. Although the reported developmental defects make it unviable for agricultural use, from a scientific perspective it might be a useful model to investigate the role that high ascorbate has in tomato physiology.

### Molecular Breeding and Genome Selection for Ascorbate Improvement

Improving fruit ascorbate content using marker-assisted selection requires prior identification of the genetic basis for natural variation of ascorbate. This can be achieved by genetic mapping and quantitative trait loci (QTL) analysis or genome-wide association studies (GWAS) in a developed mapping population, or alternatively in a diverse set of genotypes within the species, that are genotyped and phenotyped to determine molecular markers associated to specific traits (Mackay et al., 2009; Singh and Singh, 2015). Next, identified markers need to be validated for their application to select new cultivars with increased ascorbate content.

Several studies have shown that ascorbate content in fruits exhibit a quantitative inheritance, with several loci involved in ascorbate variation (Stevens et al., 2007; Zorrilla-Fontanesi et al., 2011). These studies have rarely identified the genes controlling the variation in ascorbate content, but they mark the genomic regions, and associated markers, and provide relevant information about the genetic architecture of the trait (how many loci and their quantitative contribution), as well as environmental effects. In some studies, candidate genes in those regions have been identified, with examples described below in apple, strawberry and tomato.

### Apple

In this species (*Malus domestica*), a population derived from the cultivars “Telamon” and “Braeburn” was used to identify several QTLs for ascorbate content in fruit skin and flesh on linkage groups (LG) 6, 10, and 11 in the apple genome (Davey, 2006). The QTL identified on LG10 collocates with a major QTL controlling flesh browning (Sun et al., 2014). Four regions on LG 10, 11, 16 and 17 controlling ascorbate were detected over different years in another study using the same population (Mellidou et al., 2012a). Collocations between *GGP*, *DHAR* and a nucleobase-ascorbate transporter and some of the QTL were identified. In the case of *GGP*, allelic variations in two different *GGP* genes (*MdGGP1* and *MdGGP3*) were associated with ascorbate content (QTL on LG 11 and LG 10) both in the population and across commercial apple cultivars (Mellidou et al., 2012a). In particular, differences in the expression of *MdGGP1* between fruits from high- and low-ascorbate cultivars indicate a key role for *MdGGP1* in the regulation of fruit ascorbate content (Mellidou et al., 2012a). An allele-specific SNP in this gene represents a promising tool for molecular breeding for enhanced fruit ascorbate content in apple. In the same study, the gene *MdDHAR3-3* was associated with a stable QTL for flesh browning on LG 17, suggesting that regulation of redox status of the ascorbate pool via DHAR is important for post-harvest fruit quality traits in apple. In agreement with this, transcriptomic studies revealed that prolonged post-harvest storage downregulated *DHAR* expression, resulting in the oxidation of ascorbate and thus enabling browning to occur (Mellidou et al., 2014). Therefore, besides the nutritional relevance of increasing ascorbate content in fruits, it has been shown that increased ascorbate is associated with improved post-harvest quality in fruits such as pear and apple (Davey, 2006; Mellidou et al., 2012a). For example, increased flesh browning in apple fruits is associated with the presence of a less reduced ascorbic acid pool (Davey, 2006).

### Strawberry

Strawberry (*Fragaria × ananassa*) is the fruit with the highest global production among berries, reaching a value of over nine million tons (FAOSTAT see text footnote^1^), and it typically contains high ascorbate. However, ascorbate content varies widely between strawberry cultivars and *Fragaria* species, ranging from 10 to 80 mg/100 g FW (Cruz-Rus et al., 2011; Mezzetti et al., 2016). Using a biparental population of 95 F1 progenies derived from two strawberry breeding lines, three QTL explaining a total of 45% variation were identified on LG IV-2, LG V-1 y LG VII-1 (Zorrilla-Fontanesi et al., 2011). Two of the detected QTLs were stable in different years and candidate genes were identified based in orthologous positions in the diploid *F. vesca* reference genome. The gene *FaGalUR* collocated with the position of the stable QTL on LG IV-2 and a gene encoding a myoinositol oxygenase (*FaMIOX*) was located within the stable QTL on LG V-1 (Zorrilla-Fontanesi et al., 2011), although the role of this pathway remains controversial.

*FaDHAR* and *FaGMP* collocated with the QTL detected only 1 year on LG VII-1. Recently, a transcriptomic analysis by RNA-seq in pools of progeny lines contrasting in ascorbate content derived from the same population identified differential expression of gene *MANNOSE-6-PHOSPHATE ISOMERASE 1* (*FaM6PI1*) while *FaMIOX* was not differentially expressed (Vallarino et al., 2019). The *FaM6PI1* gene was also located within the confidence interval of the major QTL detected on LG V-I, and it is highly similar to the Arabidopsis *PMI* gene that encodes the first enzyme in the SW pathway (Maruta et al., 2008). Therefore, gene *FaM6PI1* was proposed as a candidate gene contributing to the natural variation in ascorbate content in strawberry.

### Tomato

Several loci controlling ascorbate content have been detected using different populations derived from crosses between cultivated varieties (*S. lycopersicum*) and related wild *Solanum* species. Common genomic regions controlling ascorbate content have been identified on chromosomes 2, 8, 9, 10, and 12 (Stevens et al., 2007). In general, wild alleles increased ascorbate content and QTL were relatively stable across years or environments. The tomato gene *GME2* lies within the QTL interval on chromosome 9 (bin 9-J) and other candidate genes localized within QTL intervals were *MDHAR3* in bin 9-D, *GMP2* in bin 9-E, and *GLDH* in bin 10-E (Stevens et al., 2007). Further studies confirmed that this MDHAR activity was linked to ascorbate content in tomato fruits, which was found beneficial for an extended shelf life after chilling (Stevens et al., 2007). The role of MDHAR in governing ascorbate pool size was demonstrated through assessing expression and activity profiles throughout fruit ripening (Mellidou et al., 2012b). In an independent report, 163 tomato accessions were analyzed for several traits including ascorbate content by a GWAS approach and, again, significant SNPs associated to *MDHAR* were identified (Sauvage et al., 2014). All together, these reports indicate a relevant role of MDHAR in governing natural variation in ascorbate content in tomato.

Using transcriptomic analysis, a QTL detected in three trials on introgression line IL12-4 (*S. pennellii* in a *S. lycopersicum* background) was associated with up-regulation of genes involved in pectin degradation (Di Matteo et al., 2010). Further analyses of mutant variants and expression studies in introgression sublines from IL12-4 supported that pectinesterases might have a crucial role in determining ascorbate content in fruits of IL12-4 (Ruggieri et al., 2015). These studies suggested that ascorbate accumulation in IL12-4 fruits was achieved by increasing flux through the D-galacturonate pathway, as indicated above.

Recombinant Inbred Lines (RIL) have also been used to identify QTL/candidate genes linked to ascorbate content in tomato fruits. Thus, transcriptomic analyses in fruits of two groups of contrasting RILs suggested that ascorbate content co-regulates with genes involved in hormone signaling, and that they are dependent on the oxidative status of the fruit (Lima-Silva et al., 2012). Another study in tomato using the same RIL population, derived from the wild-relative *S. pimpinellifolium* TO-937, detected four QTL with a joint contribution of 42.1% to the variation of ascorbate content (Capel et al., 2015).

### Melon

A limited number of genetic studies on ascorbate have been conducted in melon, although this fruit serves a significant source of this vitamin. There is considerable variation within the species. Ascorbate content in different varieties of the most widely consumed Cantaloupe and Honeydew melons range from about 10 to 29 mg/100 g FW, with the former types having higher content than the latter (Laur and Tian, 2011). This crop has a high global production (∼31 million tons; FAOSTAT) and it is also amongst the highest productions in the European Union (∼3 million tons; Eurostat, 2017). A single QTL for ascorbate has been mapped on LG 5 using different populations (Sinclair et al., 2006; Park et al., 2009). However, low reproducible RAPD markers were used in these studies, hampering their application in breeding programs.

Overall, the number of studies identifying QTLs affecting fruit ascorbate content is still rather limited to draw conclusions on common loci across different species. In order to effectively introduce QTLs using marker-assisted selection in order to develop new fruit varieties with increased ascorbate content, loci must be validated in independent studies. Also, it is important to use additional populations and to perform the QTL analysis in different locations in order to determine QTL stability. To date, only natural variation in *GGP* and *MDHAR* alleles have been shown in independent studies to be useful in increasing ascorbate in apple and tomato, respectively (Stevens et al., 2008; Mellidou et al., 2012a). Pyramiding QTLs has the potential to increase ascorbate content, particularly in those cases when an individual QTL has a limited effect. There are already reports in which *S. lycopersicum* lines containing two chromosomal fragments from *S. pennellii* double the ascorbate content in ripe tomato fruit (Sacco et al., 2013; Rigano et al., 2014). Furthermore, with the recent establishment of high-throughput genotyping platforms, the selection of lines that include only specific genomic regions of interest will now be performed in a very efficient manner (Crossa et al., 2017).

## Conclusion and Future Perspectives

The importance of ascorbate for humans has been recently highlighted through the characterization of its role in the activity of TETs and histone demethylases. Therefore, it is important to understand the mechanisms that determine the levels of ascorbate in fruits, a major source for ascorbate in human diet. An essential role for ascorbate in plants and animals is to maintain the oxidative status in the active center of several enzymes. It is also essential for scavenging ROS produced during photosynthesis. The identification of *vtc* mutants clearly highlighted an essential role of ascorbate in oxidative stress tolerance (Conklin et al., 1996). Ascorbate has additional roles during plant growth since early reports indicated that external application of ascorbate caused a significant increase in seedling growth and effects on cell division (Hausen, 1935; Havas, 1935), although the molecular mechanisms are not completely understood.

Most of the molecular studies have been performed in the model plant Arabidopsis *thaliana*, allowing the identification of all the catalytic steps of the SW pathway. However, with the exception of the established role of GGP as a key biosynthetic control step, very little is known about the factors that determine the final content of ascorbate in different tissues.

In fruits from different species or even within the same species large differences can be observed, with fruits that show extremely high content of ascorbate such as camu (Castro et al., 2015) and acerola (Badejo et al., 2009). How these fruits can accumulate such large amounts, or what is the advantage of having such a high content of ascorbate in these fruits is not known. As previously indicated, an important aspect of ascorbate is the close interconnection between its biosynthesis and that of the non-cellulosic cell wall components, which might hamper a proper understanding of the regulation of ascorbate biosynthesis. Since degradation of the cell wall is a common process during fruit ripening, alternative pathways such as that using D-galacturonate may have an important role in the final accumulation of ascorbate in this organ. Considering all this, it is important to extend the research to ascorbate-rich fruits to identifying regulators that determine high-ascorbate accumulation. An advantage is that the high conservation of proteins of the SW pathway among plant species makes it relatively easy to identify the orthologous genes. With the current genomic tools and high throughput sequencing technology, GWAS could be a good approach to identify these components. The use of segregating populations using contrasting parental lines can also be a good choice, considering the expedition of gene identification through combination of bulk segregant analysis (BSA), high-throughput next-generation sequencing, efficient SNP arrays, mapping by sequencing approaches (Takagi et al., 2013), or global gene expression studies (Amaya et al., 2016).

The CRISPR/Cas9 technology has greatly improved our capacity to engineer targeted mutations in eukaryotic genomes (Doudna and Charpentier, 2014). In tomato, CRISPR/Cas9 has been recently used to modify quantitative trait variation in some key agronomical traits such as fruit size, inflorescence number and plant size in tomato (Rodríguez-Leal et al., 2017). In a recent report, genome editing of the uORF of *GGP* in lettuce increased the ascorbate content by 1.5-fold, leading to oxidative stress tolerance (Zhang H. et al., 2018). A similar edition of tomato *GGP1* also led to an ascorbate increase of ∼1.5-fold in leaves (Li et al., 2018). Thus, a future trend will be to use genome editing to target gene determinants in either the *cis*-regulatory elements to modify their gene expression, substrate affinity, catalytic efficiency, generation of specific alleles or targeting interacting partners to modulate the ascorbate content in fruits. All this will be further facilitated by increasing sequence replacements via homologous recombination as has been already reported in Arabidopsis through CRISPR/Cas9 (Miki et al., 2018).

## Author Contributions

MF, IA, and MB contributed to write the manuscript. MF, IA, MB, and VV contributed to the manuscript review.

## Conflict of Interest Statement

The authors declare that the research was conducted in the absence of any commercial or financial relationships that could be construed as a potential conflict of interest.

## Footnotes

1 http://www.fao.org/faostat/en/#data

2 https://ndb.nal.usda.gov/ndb/search/list
